# Peptic digestion of beef myofibrils is modified by prior marination

**DOI:** 10.3402/fnr.v57i0.20294

**Published:** 2013-05-23

**Authors:** Nash Patel, Simon J. M. Welham

**Affiliations:** Division of Nutritional Sciences, Sutton Bonington Campus, University of Nottingham, Loughborough, Leicestershire, UK

**Keywords:** marination, digestibility, acetic acid, colon cancer, myofibril, cooking, steak

## Abstract

**Background:**

Preparatory steps such as seasoning, marination, and cooking may induce changes in meat which affects the ability of the stomach to adequately digest it. This may result in peptide chains reaching the colon intact where resident bacteria ferment them resulting in the formation of putative carcinogenic phenolic by-products.

**Objective:**

In this study, we set out to determine whether peptic digestion of beef myofibrils was influenced by prior marination.

**Design:**

Cubes of sirloin stewing steak were marinated in balsamic vinegar or left untreated at 4°C overnight. Samples were oven cooked and myofibrils were extracted. Myofibrils were subject to proteolytic digestion with pepsin and digestion products analysed spectrophotometrically and with gel electrophoresis.

**Results:**

Both marination in balsamic vinegar and cooking significantly reduced the yield of myofibrils from shop-purchased beef (P<0.05). Digestion progressed in all samples as a function of time (P<0.01), varying depending on prior treatment. Marination induced resistance to the digestive effect of pepsin during the early to mid-phase of digestion, and we identified a protein band of ∼150 kDa which was protected from peptic digestion in samples which had been marinated and cooked, but not in any other groups.

**Conclusions:**

Pre-treatment of meat prior to cooking may influence specific peptides such that they become more resistant to the digestive actions of pepsin.

Meat is cooked prior to consumption to eliminate pathogenic microorganisms and to enhance its palatability. In addition, tenderisation via mechanical disruption or marination is carried out in order to aid digestion (1, 2). Marination of meat in a weak organic acid results in loosening of protein structure (3) and is particularly helpful for the preparation of meat containing large amounts of connective tissue (4). However, contradictory findings that citric acid treatment has little influence on meat tenderisation (5) to some extent calls into question whether prior marination of meat has any specific impact on its digestibility.

Interest in the digestibility of meat and processes which might influence this partly stem from the association between the products of bacterial fermentation within the colon and risk of colorectal cancer. High intakes of red meat and poor digestion of meat are positively associated with elevated risk of colonic cancer (6–10). This may in part be mediated by the production of the carcinogenic N-Nitroso Compounds (NOCs) and heterocyclic amines (11) as a result of cooking at high temperatures; however, the additional possibility remains that proteins which are incompletely digested may enable the generation of carcinogenic by-products in the large intestine. Proteins which are not readily hydrolysed by pepsin may be prevented from complete proteolysis in the small intestine. Such non-hydrolysed or partially hydrolysed proteins in the stomach have been hypothesised to ultimately become available for fermentation by colonic bacteria with the production of phenol and para-cresol (12). These are potentially mutagenic products of protein digestion and are believed to increase the risk of colonic cancer (13). The gut microflora mainly salvage energy from non-digested dietary substrates during fermentation. Consequently, phenolic compounds may be formed following the degradation of aromatic amino acids phenylalanine, tyrosine, and tryptophan (14).

Whilst previous work has focussed on the mechanisms by which proteolysis might be inhibited by prior meat treatment, here we attempt to clarify changes in the distribution of digestion products as a result of pre-treatment. We examined the impact of marination on the digestibility of shop purchased beef and the nature of the digestion products generated by pepsin digestion. We carried out this study in order to determine if prior marination might influence the type of digestion products which pass from the stomach into the small intestine.

## Methods

### Sample preparation and cooking

Sirloin stewing steak was purchased from a local supermarket and cut into small cubes weighing approximately 5 g. Samples were either marinated in balsamic vinegar (6% aceto balsamico di Modena) at a ratio of approximately 2 ml/g meat overnight (∼16 h) at 4°C prior to cooking or left covered and unmarinated at 4°C. Also, samples were either snap frozen and stored at −20°C or wrapped in silver foil and cooked in an oven (Scientific Laboratory Supplies, Hessle, Yorkshire, UK) at 100°C for 30 min. All samples were snap frozen and stored at −20°C for subsequent analysis. Storage at −20°C was limited to a maximum of 48 h. Experimental groups included unmarinated and uncooked (UMUC), unmarinated and cooked (UMC), marinated and uncooked (MUC), or marinated and cooked (MC) samples.

### Myofibril extraction

Myofibril preparation was adapted from Sante-Lhoutellier et al. (12). Briefly, samples were pulverised in liquid Nitrogen. Samples (1 g) were resuspended in 10 ml ice-cold buffer A (10 mM Tris-HCl, 1 mM EDTA, 2 mM MgCl_2_, 0.5 mM DTT, pH 7.5) and homogenised using a bench-top homogeniser (IKA, model no. Dl18BS2, Germany). Homogenates were spun at 3000 rpm for 5 min and the supernatant was carefully discarded. A further 10 ml of fresh ice-cold buffer A was then added to each of the pellets and the process was repeated. This was repeated once more before pellets were finally re-suspended in 10 ml ice cold buffer A. The concentration of myofibrillar proteins was assessed by measuring the absorbance of myofibril solution at A280 ηm (absorbance of 1 mg/ml myosin at A280 ηm=0.56) and samples were diluted to 5 mg/ml with buffer A.

### Digestion of myofibrils for SDS PAGE electrophoresis

Myofibrils (2 mg) were digested with pepsin (10 mg/ml in 33 mM glycine buffer pH 1.8) at a final concentration of 5 mg/ml; (Fisher Scientific, Loughborough, Leicestershire, UK) and incubated at 37°C for 60 min (n=3/group). Thus, all subsequent digestion protocols were conducted using myofibrils resuspended in a buffer A/glycine buffer at a ratio of 1:1. Aliquots were removed at 0 and 60 min and the reaction terminated by heating to 100°C for 5 min. Samples were examined by separation with sodium dodecyl sulphate poly acrylamide gel electrophoresis (SDS PAGE). Gels were stained with Coomassie blue and de-stained with 10% acetic acid (Fisher Scientific, Loughborough, Leicestershire, UK) over 2 days.

### Digestion of myofibrils for determination of rate of digestion

Myofibrils were prepared from meat samples (n=5/group) as described, resuspended at a concentration of 5 mg/ml and digested with pepsin (5 mg/ml final concentration) at 37°C for 60 min. Samples were taken at 0, 20, 40, and 60 min of digestion, the reactions were stopped by the addition of 30% trichloroacetic acid solution (final concentration of 15%; Fisher Scientific, Loughborough, Leicestershire, UK). Samples were centrifuged at 12,500 g for 3 min and the absorbance of the supernatant determined at 280 ηm.

### Statistical analysis

Statistical analyses were carried out using SPSS version 16 (IBM, Chicago, Illinois, USA). The effect of digestion time on the digestibility after cooking and the presence of hydrolysed peptides were tested with a repeated measures analysis, whilst measures of myofibril yield were assessed using a two-way analysis of variance.

## Results

**Fig. 1:**
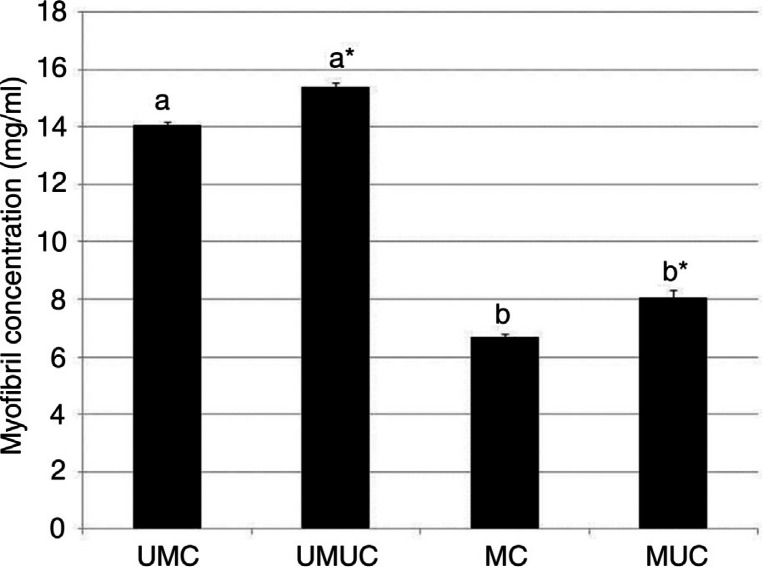
Myofibril yield. Myofibrils were prepared from beef, which had either been marinated or left untreated and cooked or retained raw. Concentration of myofibrils obtained from beef samples were treated as described are shown. Values indicated represent mean±standard error of the mean. UMUC – (n=5); UMC – (n=5); MUC – (n=5); MC – (n=5). Values were analysed using two-way ANOVA. Bars which share a letter are similar, those which do not are significantly different (P<0.01). *indicates significant difference with respect to cooked sample from the same treatment (P<0.01).

The process of marination did not appear to impact greatly on the appearance of cooked samples; however, overnight marination significantly reduced the yield of myofibrils in both cooked and uncooked samples (P<0.01; Fig. 1). Cooking itself also negatively impacted on myofibril yield (P<0.01; Fig. 1).

**Fig. 2:**
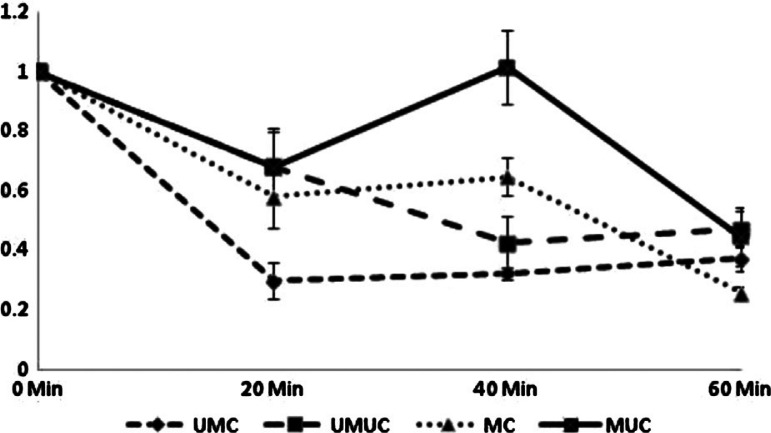
Time-course of myofibril digestion. Myofibrils were prepared from beef which had either been marinated or left untreated and cooked or retained raw. Samples were digested with pepsin to simulate stomach conditions and reactions were terminated at 0, 20, 40, and 60 min in order to assess the disappearance of trichloroacetic acid soluble proteins to indicate the degree to which samples had been digested. Values show mean change in absorbance values at 280 ηm of TCA soluble peptides (±standard error of the mean) from time 0. UMUC – (n=5); UMC – (n=5); MUC – (n=5); MC – (n=5).

Examination of the ability of pepsin to digest marinated and/or cooked beef samples revealed a marination dependent alteration of proteolysis. All samples showed a significant influence of pepsin over time (P<0.01; Fig. 2), however this varied depending upon prior treatment. Unmarinated uncooked samples showed a rapid and steady rate of proteolysis during the first 40 min of digestion (Fig. 2). After this time, the rate of proteolysis declined, with absorbance remaining stable for the final 20 min of digestion. Proteolytic degradation of unmarinated cooked samples was mostly completed within the first 20 min as evidenced by no further decline in absorbance for the remainder of the digestion procedure. Both cooked and uncooked marinated samples showed a different pattern of peptic digestion. Proteolysis progressed rapidly in the cooked marinated sample from the onset of the digestion protocol but was considerably slowed down between 20 and 40 min. However, the rapid proteolytic digestion resumed after 40 min for the remainder of the experiment. Proteolysis was minimal in marinated uncooked samples for the first 40 min of the digestion, however after this it progressed rapidly.

**Fig. 3:**
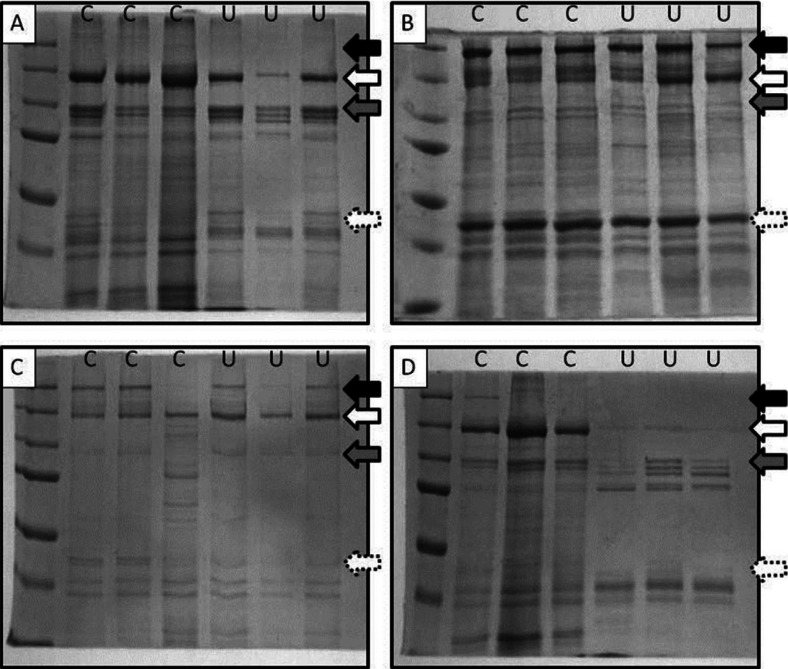
Gel electrophoresis of myofibril digests. Myofibrils were prepared from beef which had either been marinated or left untreated and cooked or retained raw. Samples were digested with pepsin to simulate stomach conditions and reactions were terminated at 0 and 60 min in order to assess the nature of the digestion products generated. Images show protein species derived from different samples. A, unmarinated samples are shown at 0 min of digestion, either cooked (C) or uncooked (U). B, marinated samples are shown at 0 min of digestion, either cooked (C) or uncooked (U). C, unmarinated samples are shown after 60 min of digestion either cooked (C) or uncooked (U). D, marinated samples are shown after 60 min of digestion either cooked (C) or uncooked (U). Solid black band – myosin heavy chain; dotted white arrow – actin; white arrow – 150 kDa of unknown proteolytic product; grey arrow – α-actinin.

In order to better understand the consequences of prior marination and/or cooking on peptic digestion of beef, we further examined the products of digestion using gel electrophoresis. We found little soluble myosin heavy chain (MHC; ∼220 kDa) from myofibril preps of unmarinated meat (Fig. 3A – solid black arrow), but a robust appearance in undigested samples subjected to marination (Fig. 3B). Digestion of marinated samples showed almost complete loss of MHC (Fig. 3D). However, in unmarinated samples, a band appeared, albeit faintly, for MHC after 60 min of digestion (Fig. 3C).

In unmarinated samples, peptic digestion generated a global reduction in all visible protein bands (Fig. 3A and 3C) and showed no influence of cooking on the distribution of bands which were diminished. Similarly, samples which had been subjected to prior marination all showed evidence of proteolytic degradation, with bands exhibiting diminished intensity after peptic digestion. This was quite pronounced for myosin (Fig. 3B and 3D – solid black band) and actin (∼42 kDa; dotted white arrow). However, we observed a band at approximately 150 kDa which appeared to increase in intensity after digestion in samples which had been subject to marination prior to cooking (Fig. 3D – white arrow). In all other samples, this band diminished as a result of peptic digestion. In addition, for all marinated samples (cooked and uncooked), α-actinin (∼103 kDa; Fig. 3D – grey arrow) appeared resistant to peptic digestion. This was not the case for unmarinated samples.

## Discussion

In this study, we examined the influence of prior marination on the ability of pepsin to digest beef myofibrils. We found that marination impacted upon the rate of digestion and at least one of the targets of pepsin. The ability to thoroughly fragment consumed meat within the stomach has importance within the context of gastrointestinal cancer, in particular colon cancer (12). Partially digested proteins which reach the colon are available for fermentation by the colonic bacterial microflora leading to the generation of compounds such as phenol and para-cresol which are themselves tumourigenic (13, 14).

Gastric digestion in humans generally takes 1–2 h (15) for small food samples macerated using the teeth with a concentration of pepsin in the stomach of 0.5–1 mg/ml (16, 17). This results in extremely thorough and efficient proteolysis with only short polypeptide chains being delivered into the small intestine where their digestion is completed to individual amino acids. The digestion protocols carried out in this study used pepsin at a final concentration of 5 mg/ml for 60 min. In our experiments, therefore, the high concentration of pepsin (five times the amount found in the stomach) used to digest very finely mechanically homogenised myofibrils may have resulted in such successful levels of peptic digestion that we may have prevented the observation of a wide array of protein species which might, under less stringent conditions, prove more resistant to degradation. Despite this, we still observed a particular protein species (∼150 kDa), which showed significant resistance to digestion in a treatment dependent manner (Fig. 3D – white arrow). Acid marination of meat post-mortem, via lactic acid injection, has been demonstrated to increase the activity of soluble cathepsins (B & L) resulting in a reduction in the quantity of intact MHC and an increase in the appearance, on western blot, of a 150 kDa band (18). The authors suggest, therefore, that the 150-kDa band represents cathepsin B+L degraded MHC. In our samples, this band was already present in unmarinated undigested samples (Fig. 3A) suggesting that a degree of proteolysis had occurred prior to experimentation. The digestion of this band was seen to be significantly inhibited in samples which had been exposed both to prior marination and cooking. This suggests that the combination of these two denaturing influences rendered this protein species resistant to the influence of pepsin and therefore potentially capable of exiting the stomach in a less digested state than it would otherwise be. Undigested and unmarinated samples also showed a very low abundance of intact MHC, whilst this appeared after digestion. It is possible that any intact MHC was so tightly complexed with itself and other proteins that it may have been inadvertently excluded from the aliquot taken at time 0. In future studies, we will take entire samples at time 0 and denature the whole sample to ensure complete coverage of protein species.

We found that the yield of myofibrils was reduced in samples which had been subject to marination (Fig. 1). This was a consistent observation as the pilot study data using only three replicates per group revealed a similar relationship with myofibril concentration (UMUC – 1.52±0.15 mg/ml; UMC – 1.51±0.06 mg/ml; MUC – 0.80±0.04 mg/ml; MC – 0.80±0.02 mg/ml; P<0.01). This reduction in the myofibril yield was an expected consequence of acidic denaturation which causes the formation of large insoluble protein complexes. Acidic denaturation of proteins will result in their precipitation in aqueous solution as seen when egg white or milk is curdled. Whilst marination is intended in part to disrupt tissue structure and thereby aid mastication and digestion, it may also be the case that several protein species may be made insoluble and therefore more resistant to digestion by prior acidic marination. This certainly appears to be the case upon examination of the rate of digestion of marinated myofibrils (Fig. 2). For both cooked and uncooked samples, it was clear that prior marination led to a temporary slowing of peptic digestion.

The apparent resistance of a specific protein species to digestion in samples which had been cooked after prior marination suggests that the process of cooking enabled the formation of alternative compounds within the meat which would otherwise not have formed. For example, carbohydrate side chains may have been added via the Maillard reaction (19) to nucleophilic amino groups only made available by the denaturing influence of the acidic marinade. It is possible that these new structures formed within the meat may inhibit the action of pepsin, thereby slowing digestion.

## Conclusions

To sum up, we have shown that myofibrils isolated from beef samples subject to an acidic marinade prior to cooking are made more resistant to peptic digestion and that such resistance may be peptide specific. Future work will aim to identify affected proteins and determine their potential contribution towards the production of tumourigenic compounds within the colon.
